# Primary mediastinal large B cell lymphoma

**DOI:** 10.1111/1759-7714.14155

**Published:** 2021-09-29

**Authors:** Yating Yu, Xifeng Dong, Meifeng Tu, Huaquan Wang

**Affiliations:** ^1^ Department of Hematology, General Hospital Tianjin Medical University Tianjin China; ^2^ Department of Lymphoma Key Laboratory of Carcinogenesis and Translational Research (Ministry of Education/Beijing), Peking University Cancer Hospital & Institute Beijing China

**Keywords:** diagnosis, drug therapy, immunotherapy, primary mediastinal large B‐cell lymphoma

## Abstract

Primary mediastinal large B cell lymphoma (PMBCL) is an aggressive large B cell lymphoma originating in the mediastinum, that mainly expresses B cell surface molecules, such as CD19, CD20, CD22, andCD79a. Clinically, they are characterized by rapidly increasing anterior mediastinal masses, which can cause compression of the surrounding tissues. The diagnosis of PMBCL mainly depends on the pathological features, imaging examination and clinical features. Currently, the most commonly used therapeutic regimens are R‐CHOP and R‐EPOCH. Radiotherapy is beneficial in some patients, but it can also lead to long‐term toxicity. The research and development of novel therapies are ongoing, and some studies have achieved encouraging results, including those conducted on chimeric antigen receptor‐modified T (CAR‐T) cell therapy and anti‐PD‐1 drugs. However, randomized controlled trials with larger sample sizes are still needed. Positron emission tomography‐computed tomography (PET‐CT) is mainly used to assess the curative effect after treatment and to guide the subsequent treatment strategy.

## Graphical abstract

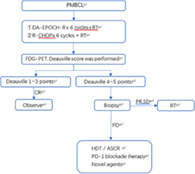

## INTRODUCTION

Primary mediastinal large B cell lymphoma (PMBCL) is a rare subtype of aggressive B cell lymphoma. It constitutes 2%–3% of all cases of non‐Hodgkin's lymphoma (NHL).^1^ PMBCL was first described in the early 1980s, when it was previously classified as a diffuse large B cell lymphoma (DLBCL) subtype.^2^ Due to its the unique clinical, histological and molecular characteristics, PMBCL has been listed as a separate type in lymphoma classification by the World Health Organization since 2016.^3^

## EPIDEMIOLOGY

The annual incidence of PMBCL is 0.4/million based on more than 400 patients diagnosed between 2000 and 2012, mostly adolescents and young adults (AYAs).^1^ It mainly occurs in patients in the age range of 30–39 years and has a predominance among females, especially among white people. Obesity, immune disorders, infection, genetics and occupational factors can lead to NHL, but no specific risk factors have been identified for PMBCL.^4^

## HISTOLOGICAL FEATURES

Most cases of PMBCL are reported to originate in the anterior superior mediastinum of the thymus region, with bulky masses, 60%–70% of which are larger than 10 cm in diameter. The lungs, pleura and pericardium are often involved. The tumor cells are usually medium to large, with abundant cytoplasm, grayish white and round or oval nuclei. Fibrosis around tumor cells leads to diffuse proliferation, leading to separation and sclerosis.^3^ PMBCL malignant cells originate from thymic B cells and express B cell antigens such as CD19, CD20, CD22 and CD79a, but do not express surface immunoglobulins. B cell transcription factors are often positive, including PAX5, OCT2, BCL6, PU1, IRF4 and BOB1.^1^, ^5^ CD30 is weakly expressed and CD15 was negative. CD23, MAL, PDL1 and PDL2 are frequently expressed.

The most common chromosomal abnormalities are +9p, +12q and +Xq. The +9p results in abnormal expansion of JAK2 located on 9p24 and abnormal regulation of the JAK–STAT pathway, overexpression of PDL1 and PDL2, and downregulation downregulation of MHC‐II and CIIA lead to the survival of PMBCL malignant cells in the thymic microenvironment.^6^ Abnormally activated JAK–STAT is involved in oncogene activation, tumor suppressor gene inactivation, abnormal cell proliferation, tumor growth and metastasis.^7^ PMBCL has a structurally activated NF‐κB pathway. Nonsense and frameshift mutations in TNFAIP3 lead to NF‐κB activation.^8^ Therefore, blocking NF‐κB, JAK–STAT pathway and PD‐1 antibody may be a potential therapeutic strategy.

**FIGURE 1:**
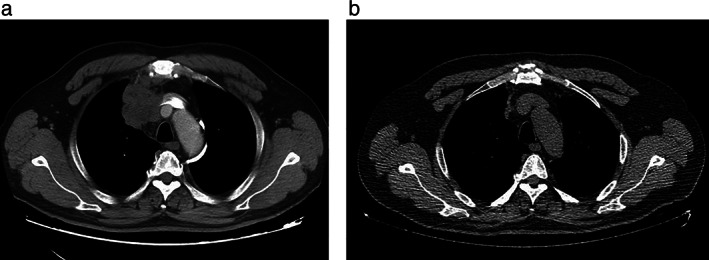
(a) Computed tomography (CT) from a male patient who presented with a primary mediastinal large B cell lymphoma. The long arrow shows the mediastinal mass. (b) CT scan from this patient after five cycles of the R‐CHOP regimen. The mediastinal mass decreased significantly

**FIGURE 2:**
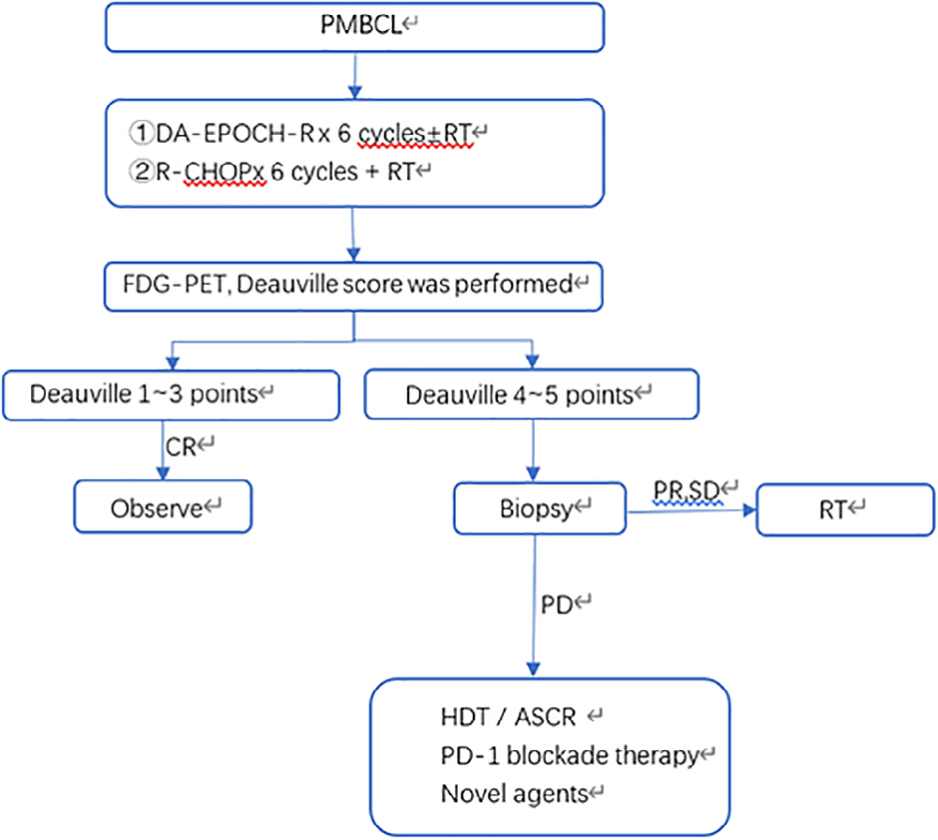
Management of PMBCL. CR, complete remission; PR, partial remission; PD, progressive disease; SD, stable disease; HDT/ASCR, high‐dose therapy followed by autologous stem cell rescue

## CLINICAL MANIFESTATION

The characteristic clinical manifestation of PMBCL is a fast‐growing mass in the anterior mediastinum, which leads to local compressed respiratory symptoms, including superior vena cava syndrome, pleural or pericardial effusion. Common symptoms include cough, dyspnea, hoarseness, dysphagia, airway or vascular damage, and B symptoms (fever, night sweats, and weight loss). Lactate dehydrogenase (LDH) is also elevated. The involvement of distant lymph nodes and bone marrow is rare.^1^ Symptoms develop rapidly, usually within a few weeks of disease onset. Eighty percent of cases are diagnosed as stage I–II.

## IMAGING

The main imaging methods for PMBCL diagnosis are computed tomography (CT), magnetic resonance imaging (MRI) and PET/CT. CT is the first‐line examination method that can detect primary lymph node enlargement and surrounding tissue. On CT, the mass usually shows low‐density features, including varying degrees of bleeding, necrosis or cystic changes.^4^ Other imaging findings include unilateral diaphragm elevation, pleural effusion and pericardial effusion. PET/CT plays an important role in the evaluation of remission status (Figure 1).

PET/CT can distinguish fibrosis, necrosis and active tumor, and is used for staging, diagnosis, and early response assessment after immunochemotherapy.^5^ A study of 113 patients showed that the 5‐year overall survival (OS) of PET/CT‐negative and PET/CT‐positive patients was 97% and 88%, respectively. In the RT era (R‐CHOP‐RT), 78% received RT treatment. In the era of PET (only patients with positive end‐of‐treatment [EOT] PET scan received RT), 28% received RT. Comparing the results of the RT and PET eras, the 5‐year OS rates were 86% and 91%, respectively. EOT‐PET was used to guide the application of consolidated RT, which reduced the usage by 64% without affecting the cure.^9^ PET/CT has a good negative predictive value, but its positive predictive value is limited. After treatment, mediastinal inflammatory tissue and tissue reactions may lead to a high false‐positive rate.^5^ LDH levels and several PET/CT findings (tumor size, presence of necrosis, and degree of F‐FDG uptake) are helpful in discriminating other diseases from PMBCL.^10^ The PET Deauville score can be used to determine which patients cannot receive consolidative radiotherapy.^1^ Deauville negative (1–3 points) patients were closely followed up with no intervention before disease progression. Deauville (4 points) patients may be due to a post‐chemotherapy inflammatory response, leading to a high false‐positive rate. The treatment of false‐positive patients is also controversial. Some investigators suggest that consolidative radiotherapy should be combined, while others believe that they can be closely observed by PET/CT and biopsy.^11^ Deauville (5 points) patients will receive radiotherapy, second‐line chemotherapy, checkpoint inhibitors, or high‐dose chemotherapy and transplantation according to the judgment of the physician. In addition to the Deauville score, metabolic tumor volume, total lesion glycolysis (TLG), and area under the curve of cumulative SUV histogram (AUC‐CSH) based on PET/CT technology might be sensitive indicators for predicting progression‐free survival (PFS) and OS. TLG is the best predictor of individual prognosis, and elevated TLG can determine an increased risk of progression in patients.^12^, ^13^ At 5 years, OS was 100% in patients with low TLG, 80% in patients with high TLG (*p* = 0.0001), and PFS was 99% and 64% (*p* < 0.0001), respectively.^14^ Moreover, baseline TLG combined with the Deauville score may show a better positive predictive value.^15^

## DIAGNOSTIC CRITERIA AND STAGING

The diagnosis and staging of PMBCL depends on pathological, imaging and clinical features. The prominent features of PMBCL are a rapidly enlarged anterior mediastinal mass, expression of B‐cell surface molecules by immunohistochemistry, pale cytoplasm and polymorphonuclear cells under light microscopy, and fibrosis around the nucleus. Hardening occurred in half of the cases. Staging was performed according to the Ann Arbor clinical staging system.

## DIFFERENTIAL DIAGNOSIS

PMBCL needs to be differentiated from DLBCL, mediastinal gray zone lymphoma (MGZL) and cHL (especially nodular sclerosis classical Hodgkin's lymphoma). It is difficult to distinguish based on clinical manifestation alone, and the final diagnosis requires a combination of morphological, immunophenotypic and imaging indicators (Table 1). CD79a, BOB1 and cycline (cHL is 100%) are helpful in distinguishing cHL from PMBCL.^16^ MAL, CD200 and CD23 can be used to identify PMBCL and DLBCL.^17^ The gene expression profile (lymph3cx) can be used to identify difficult cases.^18^, ^19^, ^20^

**TABLE 1:** The identification of PMBCL, DLBCL, cHL and MGZL

	Primary mediastinal B cell lymphoma (PMBCL)	Diffuse large B cell lymphoma (DLBCL)	Classic Hodgkin's lymphoma (cHL)	Mediastinal gray zone lymphoma (MGZL)
Origin	Thymic medullary B cells	Lymphy node germinal center, activated B cells	Lymphy node germinal center	Thymic B cells
Epidemiology				
Median age	33	55	28	35
Sex predominance	Female	Male	Varies by subtype	Male
Clinical manifestation				
Mediastinal involvement	100%	20%	80%	80%
B symptoms	<20%	50%	40%	40%
Distant metastasis	Rare	Common	Rare	Rare
Biopsy site	Biopsy of mediastinal mass	LN excisional or cutting‐needle biopsy	Bone marrow core biopsy, excisional biopsy	Biopsy of mediastinal mass
Treatment				
Treatment plan	R‐CHOP+RT, DA‐EPOCH‐R (+RT)	R‐CHOP, R‐ACVBP	ABVD+RT, BEACOPP	R‐CHOP, ABVD‐R, DA‐EPOCH‐R
Salvage therapy	HDT/ASCT, PD‐1	HDT/ASCT	HDT/ASCT	HDT/ASCT

Abbreviations: ABVD, doxorubicin, bleomycin, vinblastine, and dacarbazine; BEACOPP, bleomycin, etoposide, doxorubicin, cyclophosphamide, vincristine, procarbazine, and prednisolone; DA‐EPOCH‐R, dose‐adjusted etoposide, prednisone, vincristine, cyclophosphamide, doxorubicin, rituximab; HDT/ASCT, high‐dose therapy followed by autologous hematopoietic cell transplantation; R‐ACVBP, rituximab, doxorubicin, cyclophosphamide, vindesine, bleomycin, and prednisone; R‐CHOP, rituximab, cyclophosphamide, doxorubicin, vincristine, prednisone; RT, radiotherapy.

## TREATMENT

### First‐line treatment

At present, the National Comprehensive Cancer Network (NCCN) guidelines recommend the DA‐EPOCH‐R regimen (dose‐adjusted etoposide, prednisone, vincristine, cyclophosphamide, doxorubicin, and rituximab) with or without RT, and the R‐CHOP regimen (rituximab, cyclophosphamide, doxorubicin, vincristine, prednisone) combined with RT and R‐CHOP can bridge the ICE regimen (ifosfamide, carboplatin, etoposide), and can be combined with rituximab or RT.

In the prerituximab era, M/VACOP‐B (methotrexate/etoposide, doxorubicin, cyclophosphamide, vincristine, prednisone, and bleomycin) was better than CHOP, and combined radiotherapy could also increase the efficacy. The efficacy of the rituximab combined regimen was significantly improved. The MInT study showed that after combination with rituximab, CR increased from 54% to 80%, 3‐year event‐free survival (EFS) increased from 52% to 78%, and 3‐year OS increased from 78% to 89%. German research shows that PFS and OS in 10 years are 67% and 72%, respectively without combining with rituximab. After combining with rituximab, PFS and OS increased to 95% and 92%, respectively. Some studies have shown that the long‐term OS and/orPFS of first‐line R‐EPOCH is better, but some studies have shown that R‐CHOP‐21 has the same curative effect as R‐EPOCH, and the incidence of grade 3–4 adverse events is lower.^21^ The CR rate was higher in patients treated with DA‐R‐EPOCH than in those treated with R‐CHOP (84% vs. 70%, *p* = 0.046). Consolidative mediastinal radiation was more common after R‐CHOP than after DA‐R‐EPOCH (59% vs. 13%, *p* < 0.001). However, patients treated with DA‐R‐EPOCH were more likely to experience treatment‐related diseases, such as neutropenic fever and acute toxicities (Table 2).^22^

### Radiotherapy

PMBCL is a radiosensitive disease, and consolidation mediastinal radiotherapy can improve its curative effect.^5^ However, PMBCL patients are young and have a long life expectancy, therefore, the long‐term toxicity of radiotherapy should be considered. Radiotherapy may increase the risk of a second malignancy, especially breast cancer and cardiotoxicity.^1^ Based on the limited published data, it is strongly recommended that the R‐CHOP regimen be combined with radiotherapy. However, for the DA‐EPOCH‐R regimen, EOT‐PET is recommended to guide RT.^9^ It has been confirmed that the use of dose‐intensive therapy without radiotherapy can also achieve a good curative effect.^5^ Moreover, some studies have shown that R‐CHOP combined with RT and DA‐EPOCH‐R regimen without RT can achieve similar efficacy, but whether high‐dose chemotherapy can replace RT remains to be discussed.^33^

**TABLE 2:** Comparison of different treatment schemes for PMBCL

	Treatment plan	Number of patients	CR rate	PFS	OS	Reference
Before rituximab	CHOP (6) MACOP‐B (15)	21	0 87%	90% (3 years)		23
	CHOP (14) MACOP‐B (15)	29	36% 73%	72% (3 years)		24
	MACOP‐B + RT	92	87%	84% (5 years)	88% (5 years)	25
After rituximab administration	M/VACOP‐B (47) R‐CHOP (19) CHOP (67)	153	77%	69% (5 years)	87% (5 years) 81% (5 years) 71% (5 years)	26
	R‐M/VACOP‐B + RT	45	80%	88% (5 years)	80% (5 years)	27
	CHOP‐like (43) R‐CHOP‐like (44)	87	32.6% 52.3%	52% (3 years) 78% (3 years)	78.2% (3 years) 88.5% (3 years)	28
	R‐CHOP (76) CHOP (45)	121	Not analyzed	81% (5 years) 48% (5 years)	89% (5 years) 69% (5 years)	29
	R‐CHOP (45) CHOP (35)	80	13% 37%	95% (10 years) 67% (10 years)	92% (10 years) 72% (10 years)	30
	R‐CHOP	63	71%	68% (5 years)	79% (5 years)	31
	R‐MACOP‐B	74	82.4%	87.6% (10 years)	82% (10 years)	32
	R‐CHOP (56) DA‐EPOCH‐R (76)	132	69.6% 84.2%	76% (2 years) 85% (2 years)	89% (2 years) 91% (2 years)	22

### Treatment of relapsed/refractory PMBCL

Disease recurrence usually occurs in the early stage, and recurrence‐refractory patients account for 10%–30% of the total number of cases.^34^ High‐dose therapy followed by autologous hematopoietic cell transplantation (HDT/ASCT) and immune checkpoint blockade therapy are effective treatments for relapsed or refractory PMBCL.^35^

The total effective rate of HDT/ASCT was 77.2% (complete remission, 63.6%). The median follow‐up time was 53.5 months. The 4‐year OS and PFS rates were 70% and 61%, respectively. The 4‐year OS rates of patients with relapse and primary refractory diseases were 73% and 65%, respectively.^34^ The 2‐year OS and PFS of allo SCT were 45% and 39%, respectively. Allo SCT treatment has high transplant‐related mortality.^36^ Moreover, it may produce durable remission in a proportion of patients with treatment‐sensitive disease before transplantation.^37^

The most promising target of immune checkpoint blockade for PMBCL is PD‐1. The US Food and Drug Administration (FDA) has approved the use of pembrolizumab for patients with RR PMBCL. PD‐1 blockade therapy enhances the immune response mainly by reactivating the existing antitumor T cells in situ, rather than activating de novo antitumor T cells, which is defined as a checkpoint blockade therapy (CBT)‐responsive lymphoma (i.e., inflamed lymphomas), and its lymphoma environment is enriched in infiltrating immune cells.^38^ In addition to the overexpression of PDL1 and PDL2, there are two aspects which give immune checkpoint inhibitors a good therapeutic potential. Pembrolizumab is an anti‐PD‐1 immune checkpoint inhibitor that has been approved by the FDA for PMBCL patients with progression after two or more treatment regimens. It has a high response rate, persistent activity, and manageable safety.^39^, ^40^, ^41^ In the KEYNOTE‐013 study, 21 patients with relapsed or refractory PMBCL were treated with pembrolizumab. The objective response rate (ORR) was 48%, including seven CR and three PR. In 81% of patients, the lesions shrank. A phase 2 trial showed that in 53 patients, the ORR was 45%, including 7 CR (13%) and 17 PR (32%).^41^ PD‐1 monoclonal antibodies combined with chemotherapy can further improve efficiency. Camrelizumab combined with gemcitabine, vinorelbine, and pegylated liposomal doxorubicin (GVD) was used to treat 27 cases of relapsed or refractory PMBCL. The data showed that the ORR was 74%, the CR was 56%, the median response time was 1.7 months, and 78% showed tumor shrinkage at the first evaluation.^42^ The ORR of the PD‐1 antibody nivolumab combined with brentuximab vedotin for the treatment of relapsed or refractory PMBCL was higher, up to 73%, but the number of adverse events were more than that of penbrolizumab alone (83%). A total of 16 patients (53%) had grade 3 to 4 treatment‐related adverse events; the most common were neutropenia (*n* = 9), thrombocytopenia (*n* = 3), and peripheral neuropathy (*n* = 3).^43^ The combination of platinum‐based chemotherapy and PD‐1/PD‐L1 inhibitors has also shown therapeutic potential.^44^

### Novel therapies

The potential targets of PMBCL therapy include specific cell surface markers, cell signal transduction pathways, programmed death ligands, etc. At present, several new drugs for PMBCL therapy are undergoing clinical trials, some of which have achieved encouraging results.

#### Chimeric antigen receptor T (CART) cell therapy

Anti‐CD19 CAR‐T cell therapy shows good therapeutic prospects. CD19 is a common target antigen in hematological malignancies, and is commonly found on B cells.^7^ ZUMA‐1included 101 patients with RR‐DLBCL, PMBCL (*n* = 8) and transformed FL. Among all patients in 1 year, ORR was 82%, CR was 54%, with a median follow‐up of 15.4 months, 42% of the patients continued to have a response, with 40% continuing to have a complete response, and 18 month survival was 52%.^45^ The longest follow‐up time of anti‐CD19 CAR‐T cell therapy for PMBCL/DLBCL was 97 months. The peak level of CAR‐T cells in peripheral blood is related to the response, but the level of CAR‐T cells 28–56 days after infusion is not related to the response, and the degree of persistence of CAR‐T cells required for remission of persistent lymphoma is unclear.^46^ CAR‐T cell therapy is associated with cytokine release syndrome, and nerve events and treatment‐related death may occur,^35^ but the incidence of severe cytokine release syndrome and nervous system events is low. In the transcend NHL 001 study, 344 cases of RR‐DLBCL (including 15 cases of PMBCL) were treated with anti‐CD19 CAR‐T. The effective and CR rates were 73% and 53%, respectively. Grade 3 and above cytokine syndrome and neurological reactions were 2% and 10%, respectively.^47^

#### Brentuximab vedotin

The anti‐CD30 antibody drug conjugate brentuximab vedotin (BV) has been approved for the treatment of relapsed or refractory HD and anaplastic large cell lymphoma (ALCL). PMBCL also expresses CD30, but its expression level is low. In a phase II trial, the effective rate was 17% in six cases of PMBCL and three cases of SD. The reaction was not related to the quantitative expression of CD30 on tumor cells.^48^ In another study, 15 cases of relapsed PMBCL were treated with BV, with a total effective rate of 13.3% (2/15), two cases of PR, one case of SD and 12 cases of PD.^49^ Although BV single drug therapy results are poor, when combined with immunochemotherapy, good results have been reported. A total of 29 cases of PMBCL were treated with BV combined with R‐CHP. The effective rates were 100%, 86% and 14% PR with 2‐year PFS of 85%, and 2‐year OS of 100%.^50^

#### Bispecific T cell engager (BiTE) antibody

BiTE can simultaneously bind two different antigens. Blinatumomab is a CD19/CD3 BiTE double antibody that is used to treat Philadelphia chromosome‐negative acute B‐lymphoblastic leukemia. Blinatumomab connects CD3 + polyclonal T cells to CD19 + B cells, induces T cell activation, and T cell‐mediated B‐cell lysis. Single drug blinatumomab has been reported to show antilymphoma activity in relapsed or refractory NHL, and PMBCL data needs to be studied further.^51^

#### BKT inhibitors

Ibrutinib, a Bruton tyrosine kinase inhibitor related to the NF‐κB pathway, has also been used in combination with R‐ICE in RR‐DLBCL, including four patients with PMBCL. In four cases of PMBCL patients, ORR was 100%, all showed PR, showing good curative effect, but there were not enough cases in this study.^52^

#### JAK inhibitors

Abnormal activation of the JAK–STAT pathway in PMBCL malignant cells leads to cell proliferation, survival, angiogenesis and transcription of immune target genes. The role of JAK inhibitors in PMBCL has also been studied, and ruxolitinib is ineffective (Figure 2).^53^

## PROGNOSIS

The 5‐year survival rate of patients with PMBCL is approximately about 85%. The International prognostic index (IPI), age, LDH, stage, Ki‐67 proliferation index and SUVmax of PET‐CT are significantly correlated with survival rate.^33^ MUM1 expression and higher peripheral blood lymphocyte/monocyte ratio are significantly correlated with a better survival rate.^35^

In conclusion, PMBCL is a rare subtype of aggressive B‐cell lymphoma, which is more common in adolescents and young adults, mainly women. Treatment should consider maximum efficacy and minimize long‐term toxicity. R‐CHOP + RT and DA‐EPOCH‐R ± RT are effective first‐line choices for PMBCL. Autologous or allogeneic SCT after high‐dose chemotherapy is an effective treatment strategy for relapsed or refractory PMBCL. Following immunochemotherapy, FDG‐PET is used to evaluate the therapeutic effect and decide whether radiotherapy should be used. Some new therapies have shown good prospects, and clinical trials are currently in progress.

## CONFLICT OF INTEREST

All authors declare no conflict of interest.
